# Fluctuation of bone turnover markers’ levels in samples of gingival crevicular fluid after orthodontic stimulus: a systematic review

**DOI:** 10.1186/s13643-021-01860-w

**Published:** 2022-01-04

**Authors:** L. Kakali, I. Giantikidis, I. Sifakakis, E. Kalimeri, I. Karamani, E. Mavrogonatou, D. Kloukos

**Affiliations:** 1 Department of Orthodontics, School of Dentistry, National and Kapodistrian University of Athens, Athens, Greece; 2 Department of Orthodontics and Dentofacial Orthopedics, 424 General Military Hospital, Thessaloniki, Greece; 3 Department of Orthodontics and Dentofacial Orthopedics, 251 Hellenic Air Force & VA General Hospital, Athens, Greece; 4 Laboratory of Cell Proliferation and Ageing, Institute of Biosciences & Applications, National Centre for Scientific Research “Demokritos”, Athens, Greece; 5 Department of Orthodontics and Dentofacial Orthopedics, School of Dental Medicine, University of Bern, Bern, Switzerland

**Keywords:** Orthodontic tooth movement, Gingival crevicular fluid, Bone turnover markers

## Abstract

**Background:**

The aim of the present study was to provide an overview of gingival crevicular fluid (GCF) bone turnover markers (BTMs) concerning the physiology of orthodontic tooth movement (OTM) and assess their potential contributions to regulating bone remodeling, that could prove useful in designing future approaches to modulating orthodontic tooth movement.

**Methods:**

Multiple electronic databases (MEDLINE/PubMed, Ovid MEDLINE, Ovid Embase, LILACS, and Cochrane Library) were searched up to October 1st, 2020. Randomized controlled trials (RCTs), controlled clinical trials, observational studies of prospective and retrospective designs, and cross-sectional studies reporting on levels of BTMs in GCF were eligible for inclusion. The quality of the included RCTs was assessed per the revised Cochrane risk of bias tool for randomized trials (RoB 2.0), whereas the risk of bias of the included cohort studies was assessed using the Risk Of Bias In Non-randomized Studies of Interventions tool.

**Results:**

Five RCTs, 9 prospective cohort studies, and 1 cross-sectional study fulfilled the inclusion criteria. The risk of bias was deemed as high for the RCTs and 4 of the prospective studies and moderate for the rest of the studies. The following biomarkers for bone formation were assessed: bone alcaline phosphatase (BALP), alcaline phosphatase (ALP), and osteocalcin (OC). For bone resorption, the following BTMs were assessed: deoxypyridinoline (DPD) and pyridinoline (PYD), N-terminal telopeptide (NTX), osteopontin (OPN), and tartrate-resistant acid phosphatase (TRAP). The follow-up period ranged mainly from baseline to 45 days, although one study had an expanded follow-up period of up to 16 months. The results of the included studies comparing different BTMs were heterogeneous and qualitatively reported.

**Conclusions:**

Current evidence continues to support the potential for BTMs to provide clinically useful information particularly for adjusting or standardizing the orthodontic stimulus. The present systematic review has retrieved studies of high, overall, risk of bias, and has unveiled a substantial clinical and methodological heterogeneity among included studies. Further data of the relationships between the clinical assays and the physiological or pre-analytical factors contributing to variability in BTMs’ concentrations are required.

**Systematic review registration:**

CRD42020212056.

**Supplementary Information:**

The online version contains supplementary material available at 10.1186/s13643-021-01860-w.

## Background

Orthodontic tooth movement (OTM), as a biological process, encompasses a series of histological and biochemical reactions [1]; these lead to bone and tissue remodeling, which involves the dental pulp, periodontal ligament (PDL), alveolar bone, and gingiva. Force application disrupts the equilibrium that exists between bone formation and bone resorption, resulting in more bone resorption on the pressure side and more bone formation on the tension side during OTM. The mechanical stimulus causes inflammatory responses in periodontal tissues, alterations in blood flow, as well as formation and release of various chemical mediators [2].

A reflection of these phenomena can be found in the gingival crevicular fluid (GCF) of moving teeth, with significant elevations in the concentrations of its components. The noninvasive nature and the convenience of repetitive sampling of GCF are considered of great importance for identifying the periodontal changes followed by orthodontic force application [3].

There are three main methods of collecting GCF: (a) the gingival washing technique, which consists of perfusing the GCF with an isotonic solution of fixed volume; the fluid collected represents a dilution of crevicular fluid, containing cells and soluble constituents, as plasma proteins; (b) insertion of capillary tubes, with specific diameter, into the entrance of the gingival crevice; the fluid then migrates into the tube by capillary action. (c) The most common method, however, of collecting GCF is with the use of absorbent sterilized paper strips. The paper strips are inserted into the gingival crevice and left in situ for 5 to 60 s to allow the GCF to be adsorbed by the paper [4].

Several substances can be collected from GCF and are considered biomarkers. The term ‘biomarker’ depicts a substance that is measured and evaluated objectively as an indicator of normal biological processes, pathological processes, or pharmacological responses to a therapeutic intervention [3].

Numerous protein or protein derivative biomarkers are released during bone remodeling by osteoblasts or osteoclasts and are generally described under the term of bone turnover markers (BTMs) [5]. BTMs largely represent products of bone proteins, particularly type I collagen which undergoes substantial post-translational modification during synthesis of new bone. Other BTMs are products of bone cells, reflecting the number of particular cells within the bone environment at any given time [6].

BTMs have been studied for over 30 years, and they are separated into two groups: markers of bone formation (including among others N-terminal collagen type I extension pro-peptide (PINP), osteocalcin (OC), and bone alkaline phosphatase (BALP)) and markers of bone resorption (including collagen I degradation products such as C-terminal cross-linking telopeptide of type I collagen (CTX) and N-terminal telopeptide of type I collagen (NTX)) [7]. However, even though BTMs have been assessed in basic research, they are not widely implemented in clinical orthodontic practice. The primary challenge to their adoption in routine practice has been the poor within-subject and between-lab reproducibility [7].

In orthodontics, biomarkers related to bone turnover may introduce new possibilities for understanding bone growth and remodeling. Knowledge of the ongoing process occurring in periodontal tissues during orthodontic and orthopaedic therapies can lead to proper choice of mechanical loading with the aim of shortening the period of treatment and avoiding adverse effects associated with orthodontic treatment [8].

The aim of the present study was to provide insights into possible GCF BTMs concerning the physiology of orthodontic tooth movement and assess their potential contributions to regulating orthodontic processes that could prove useful in designing future approaches to modulating orthodontic tooth movement.

## Materials and methods

### Reporting format

The latest (2021) Preferred Reporting Items for Systematic Reviews and Meta-Analyses (PRISMA) were adopted throughout the process of the present systematic review [9, 10].

### Population (P), intervention (I), comparison (C), outcomes (O), and study design (PICOS)

Participants (Population):orthodontic patients of any age and sex.

Intervention: any type of removable or fixed orthodontic appliance resulting in OTM.

Comparisons: any control group was accepted (i.e. untreated group, contralateral sides in split-mouth design, control group with different types of orthodontic activations (i.e. force applied and constant or increasing forces)).

Outcomes: quantitative and qualitative analyses of bone remodeling biomarkers detected in GCF; measurement units were nanograms per microlitre (ng/μl), international units per microlitre (IU/μl), units per milligram (U/mg), picograms per millilitre (pg/ml), and milliunits per sample (mU/sample). Receptor activators or mediators not considered as exclusive bone remodeling biomarkers were excluded. Follow-up: All observation periods were accepted.

Study design: Any study design was considered eligible for inclusion in this review, including randomized clinical trials (RCTs), non-randomized or quasi-randomized controlled trials, and prospective and retrospective studies.

Exclusion criteria: animal and in-vitro studies, case reports or studies reporting less than 5 patients, studies including patients with systemic disorders affecting periodontal and orthodontic therapy, preclinical studies/ abstracts/ letters to editors/ narrative reviews, insufficient/unclear information not allowing data extraction, and no author response to inquiry email for data clarification.

### Search strategy

Detailed search strategies were developed and appropriately revised for each database, considering the differences in controlled vocabulary and syntax rules by the last author (DK). No language or publication date restrictions were applied.

#### Electronic search

On October 1st, 2020, we updated and searched the following electronic databases to find reports of relevant published studies:

- The Cochrane Central Register of Controlled Trials (CENTRAL) (up to October 1st, 2020);
- MEDLINE (PubMed) (1946 to September Week 4, 2020);
- Ovid MEDLINE (in-process & other non-indexed citations, October 1st, 2020);
- Ovid Embase (1974 to October 1st, 2020);
- LILACS (1982 to October 1st, 2020)

The search strategy of all databases is shown in Additional files 3 & 4.

#### Unpublished literature search

In order to further identify potential articles for inclusion, grey literature was searched in the register of clinical studies hosted by the US National Institutes of Health (http://www.clinicaltrials.gov), the multidisciplinary European database (http://www.opengrey.eu), the National Research Register, and Pro-Quest Dissertation Abstracts and Thesis databases (https://about.proquest.com).

#### Manual search

Experts in the field were contacted in order to find additional literature that might be relevant. The reference lists of all identified eligible studies and other published systematic reviews were hand-searched in order to identify further eligible studies. No language or publication time restrictions were applied.

### Study selection

Study selection was performed independently and in duplicate by the first 2 authors of the review (LK and IG), who were not blinded to the identity of the authors of the studies, their institutions, or the results of their research. Study selection procedure comprised of title-reading, abstract-reading, and full-text–reading stages. After exclusion of non-eligible studies, the full report of publications considered by either author as eligible for inclusion was obtained and assessed independently. Disagreements were resolved by discussion and consultation with the third author of the review (IS). A record of all decisions on study identification was kept.

### Data collection

The first two authors (LK and IG) performed data extraction independently and in duplicate. Disagreements were resolved by discussion with the last author (DK). Specifically designed Excel collection forms were used to record the desired information. Data extraction was piloted in five random included papers between the two first authors. The following data were collected: author/title/year of study, design of study, number/age/gender of patients recruited, type of orthodontic treatment, method of GCF collection, tooth site of GCF collection, control group, observation period (follow-up of patients), changes of biomarkers in GCF, biological consequence, and clinical significance.

If stated, the sources of funding, trial registration, and publishing of the trial’s protocol was recorded. This information was used to aid assessment of heterogeneity and the external validity of the included studies. In case of missing data, it was attempted to contact the corresponding author. Studies without enough data for meta-analyses were kept in the systematic review, but excluded from the meta-analyses.

### Quality assessment

The methodological quality of all included studies was assessed by the first two review authors (LK and IG) independently and in duplicate. For interventional, randomized controlled trials (RCTs), the Risk of Bias 2.0 tool was used [11]. For interventional, non-randomized controlled trials the Risk Of Bias In Non-randomized Studies of Interventions (ROBINS-I) tool was used [12]. For cross-sectional studies, the Newcastle- Ottawa Scale, adapted for this design, was implemented [13]. The overall quality of evidence (i.e. the strength of clinical recommendations) from the direct analysis was rated using the Grades of Recommendations, Assessment, Development and Evaluation (GRADE) approach [14]. Concerns were resolved by discussion with the 3rd author (IS).

### Data analysis

Meta-analyses would have been conducted if included studies reported similar interventions and comparable outcomes in homogeneous population (i.e. in the case of limited heterogeneity). For continuous variables, mean differences and standard deviations would be used to summarize the data from each study. For dichotomous data, number of participants with events and total number of participants in experimental and control groups would be analyzed. Regarding meta-analysis for dichotomous data, risk ratios and their 95% confidence intervals (Cls) would be calculated. For continuous data, mean differences and 95% Cls would be calculated.

### Heterogeneity

Clinical and methodological heterogeneity were assessed by examining the characteristics of the studies, the similarity between the types of participants, the interventions, and the outcomes as specified in the inclusion criteria for considering studies for this review. Statistical heterogeneity would have been assessed using a Chi^2^ test and the I^2^ statistic.

### Assessment of reporting bias

Reporting biases arise when the reporting of research findings is affected by the nature or direction of the findings themselves [15]. Potential reporting biases including publication bias, multiple (duplicate reports) publication bias, and language bias in this review were reduced by conducting an accurate and at the same time a sensitive search of multiple sources with no restriction on language. A search for ongoing trials was conducted, too. In the presence of more than 10 studies in a meta-analysis, the possible presence of publication bias would have been investigated for the primary outcome.

### Subgroup analyses/ sensitivity analysis

As no sufficient data existed, subgroup analyses based on study characteristics or sensitivity analysis based on the risk of bias were not conducted.

### Unit of analysis issues

We anticipated that some of the included studies presented data from repeated observations on participants, which could lead to unit-of-analysis errors. In such cases, we followed the advice provided in section 9.3.4 of the Cochrane Handbook for Systematic Reviews of Interventions [15]: we would either define several outcomes to reflect short- and long-term follow-ups, based on different time periods, and perform separate analyses, or we would select a single time point and analyze only data at this time for studies in which it is presented.

## Results

### Description of studies

**Fig. 1:**
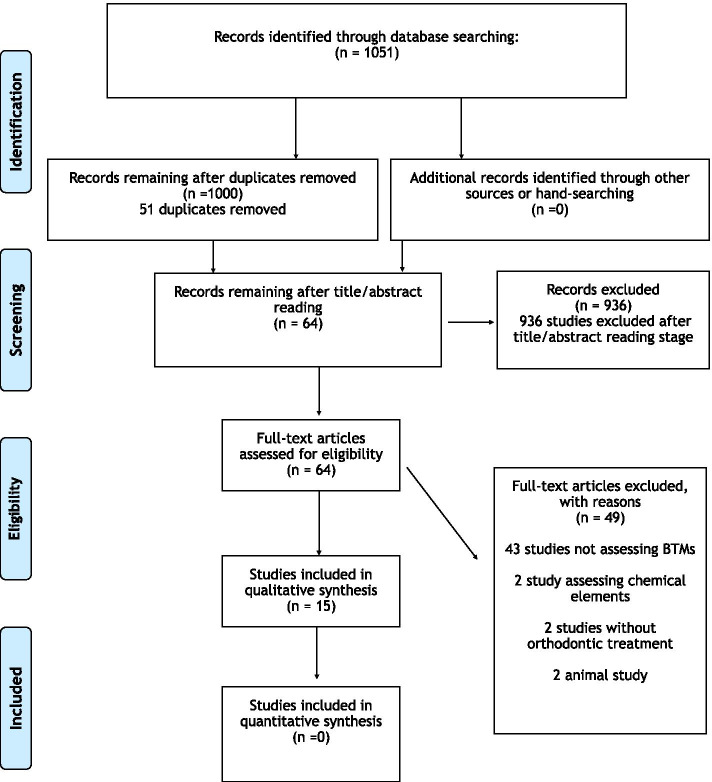
Flow diagram of studies’ selection

In total, 1051 studies were identified from the electronic searches as relevant. After exclusion of all duplicates and assessment of the title and abstract of the reports, 64 studies were considered eligible for inclusion in this review. Out of the 64 studies, another 49 studies were excluded after full-text assessment, leaving 15 studies fulfilling the inclusion criteria (Additional file 5). Five were RCTs [16–20], 9 were prospective, non-randomized studies [21–29] and 1 was of cross-sectional design [30] (Table 1). The process of final study inclusion in this review is presented in Fig. 1.

**Table 1:** Characteristics of included studies

Authors Study design	Study title	Sample size/sex/age	Health status/ drug intake	Type of orthodontic treatment	Observation period	Method of GCF collection	Tooth site of collection	Control group	Biomarker assessed
Castroflorio et al. 2017 [16] RCT/ Split mouth	Biochemical markers of bone metabolism during early orthodontic tooth movement with aligners	10 (5 F, 5 M) Mean age 22.3 ± 3.3 years	No anti-inflammatory or antibiotic therapy in the previous 6 months	Aligners (Invisalign)	• 1 h before placement • 1 h after aligner placement • 7 days after • 21 days after	Periopaper strips	Distobuccal and mesiobuccal sites of second molar under distalization	Contralateral second molar	Osteopontin (OPN)
Al Swafeeri et al. 2015 [17] RCT/ Split mouth	Crevicular alkaline phosphatase activity during the application of two patterns of orthodontic forces	12 (7 F, 5 M) Mean age 17.5 ± 2.4 years	Healthy medical status/ drug intake not reported	Fixed appliances/ extraction of 1st premolar/ distalization of canine	Baseline (before the activation) and every week for 3 weeks after the initial activation	Paper strips	Constant orthodontic force 150cN for 3 weeks mesial side of maxillary canine and distal site of maxillary first molar	Increasing orthodontic force 50 cN throughout the first week, 100 cN throughout the second week and 150 cN the third week.	Alcaline phosphatase (ALP)
Wahab et al. 2014 [18] RCT/ Split mouth	Enzyme activity profiles and ELISA analysis of biomarkers from human saliva and GCF during orthodontic tooth movement using self-ligating brackets	19 (14 F, 5M) Between 16 and 28 years old	No drug intake during the study or 1 month before	Fixed appliances/ extraction of 1st premolar/ distalization of canine	Baseline and every week for 5 weeks after the initial application	Periopaper strips	100-g force on tested canine	150-g force on contralateral canine	Tartrate-resistant acid phosphatase (TRAP) Alkaline Phosphatase (ALP)
Barbieri et al. 2013 [19] RCT/ Split mouth	Biochemical markers of bone metabolism in GCF during early orthodontic treatment	10 (5 F, 5 M) Aged from 22–29 years	No anti-inflammatory or antibiotic therapy in the previous 6 months	Separators	• Baseline • 24 h • 7 days after the placement of separators	Periopaper strips	Mesiobuccal and distobuccal sides of tested molars	Mesiobuccal and mesiolingual of contralateral molars	Osteopontin (OPN)
Kalha et al. 2010 [20] RCT	Redefining orthodontic space closure: sequential repetitive loading of the periodontal ligament—a clinical study	10 (6 F, 4 M) Mean age 20.6 ± 3.2 years	Healthy medical status/ drug intake not reported	Fixed appliances/ extraction of 1st premolar/ distalization of canine	• 1 h before • 1 h after the activation • On days 7, 14, 21, and 28	Sterile paper strips	Distogingival margin of the four canines (5 patients with hycon-screw)	Distogingival margin of the four canines (5 patients with active tie-backs)	Alcaline phosphatase (ALP)
Bitra et al. 2017 [21] Prospective	Gingival crevicular fluid turnover markers in premenopausal vs. postmenopausal women receiving orthodontic treatment	25 F Postmenopausal (mean age 57 years)	No drug intake 1 month before the study	Fixed appliances	• Baseline • 24 h	Periopaper strips	NA	25 F Premenopausal (mean age > 30 years)	Osteopontin (OPN)
Smuthkochorn et al. 2017 [22] Prospective	Gingival crevicular fluid bone turnover biomarkers: how postmenopausal women respond to orthodontic activation	16 F Postmenopausal (mean age 63 years)	No drug intake 1 month before the study	Fixed appliances	• Baseline • 24 h	Periopaper strips	2 Anterior and 2 posterior teeth	12 F Premenopausal (mean age 32 years)	Osteopontin (OPN)
Yang et al. 2014 [23] Prospective	Preliminary study on the best-exerted force chance in the female menstrual cycle	12 F Aged 18–28 years	Not reported	Fixed appliances/ extraction of 1st premolar/ distalization of canine	• Baseline • 15 days • 30 days • 45 days after	Paper strips	Distal site of canine that is distalized	NA	Osteocalcin (OC)
Alfaqeeh et al. 2011 [24] Prospective	Osteocalcin and N-telopeptides of type I collagen marker levels in gingival crevicular fluid during different stages of orthodontic tooth movement	20 (10 F, 10 M) Aged 15–25 years	No regimen or antibiotic therapy the last 3 months	Fixed appliances/ extraction of 1st premolar/ distalization of canine	• 1 h, 1 day, 7, 14, and 21 days after application	Periopaper strips	Buccal and palatal, mesial, and distal sites of tested canines	Contralateral canine with fixed appliance but not activated	Osteocalcin (OC) N-telopeptides (NTX)
Batra et al. 2006 [25] Prospective	Alkaline phosphatase activity in gingival crevicular fluid during canine retraction	10 F Aged 12–21 years	Healthy medical status/ drug intake not reported	Healthy medical status/ drug intake not reported	• Baseline (before the activation) • Immediately after • 1 day • 7 days • 14 days • 21 days after	Micropipettes of 1 μl capacity	Mesial and distal sites of tested canine	Contralateral canine (no forced applied)	Alcaline phosphatase (ALP)
Isik et al. 2005 [26] Prospective	Bone marker levels in GCF during orthodontic intrusive tooth movement. A preliminary study.	9 (5 F, 4 M) Mean age: 14.6 ± 2.08 years	Not reported	Fixed appliances/ intrusion of 1st premolar	• 1 h • 24 h • 168 h after 1st activation • 22nd day • 28th day after 2nd activation	Periopaper strips	Mesiobuccal, distobuccal, palatinal crevicular region of maxillary first premolars	NA	Deoxypyridinoline (DPD) Osteocalcin (OC) N-telopeptide (NTX) Bone alkaline phosphatase (BALP)
Perinetti et al. 2004 [27] Prospective	Longitudinal monitoring of sub-gingival colonization by *Actinobacilllus actinomycetemcomitans* and crevicular alkaline phosphatase and asparate aminotransferase activities around orthodontically treated teeth	21 (11 F, 10 M) Mean age 17.1 ± 3.3 years	No drug intake 1 month before the study	Fixed appliances/ extraction of 1st premolar/ distalization of canine	• Baseline • On day 28	Standardized sterile paper strips	Mesial and distal sites of experimental canine	Mesial and distal sites of contralateral and antagonist canine	Alcaline phosphatase (ALP)
Perinetti et al. 2002 [28] Prospective	Alkaline phosphatase activity in GCF during human orthodontic tooth movement	16 (10 F, 6 M) Mean age 15.5 ± 3.5 years	No drug intake 1 month before the study	Fixed appliances/ distal retraction of 1st molar	• 1h after activation • 1, 2, 3, and 4 weeks after	Sterile paper strips	Mesial & distal sites of maxillary 1st molar	Contralateral molar with fixed appliance but not activated and antagonist molar without appliance	Alcaline phosphatase (ALP)
Griffiths et al. 1998 [29] Prospective	Evaluation of osteocalcin and pyridinium crosslinks of bone turnover in gingival crevicular fluid during different stages of orthodontic treatment	20 (12 F, 8 M) Mean age 13.5 years	Not reported	Fixed appliances/ extraction of 1st premolar/ distalization of canine	T1 prior to orthodontic appliance, T2 post appliance fit but prior to activation, T3 during active retraction, T4 during retention	Paper strips	Distal surface of maxillary canines	NA	Osteocalcin (OC) Pyridinoline (PYD) Deoxypyridinoline (DPD)
Insoft et al. 1996 [30] Cross-sectional	The measurement of acid and alkaline phosphatase in GCF during orthodontic movement	30 (sex NA) Aged 8–12 years	Not reported	3 Groups: 1) Bionator 2) Headgear 3) Control group	One collection time point	Periopaper strips	Mesial and distal sites of 1st molars	Control group	Acid and alcaline phosphatase (ALP)

### Quality assessment

#### RCTs

The summary of methodological quality of the 5 included RCTs assessed on the basis of the Cochrane risk of bias tool is shown in Table 2. All were evaluated to be at high risk of bias [16–20]. This was mainly attributed to bias arising from the randomization process and bias arising in measurement of the outcome. Blinding of the clinicians, patients, and assessors was not universally possible due to the nature of the interventions, but the possibility of bias could not be excluded. Losses to follow-up were appropriately described, and there was no evidence of selective outcome reporting and other biases (Table 2).

**Table 2:** Risk of bias of included RCTs

	Author/ year	Bias arising from the randomization process	Bias due to deviations from the intended interventions	Bias due to missing outcome data	Bias in measurement of the outcome	Bias in selection of the reported result	Overall bias
1	Castroflorio et al. 2017 [16]	Authors' judgement: **high risk** Support for judgement: no details provided about randomization and allocation concealment processes	Authors’ judgement: s**ome concerns** Support for judgement: carers and trial personnel aware of participants’ assigned intervention	Authors’ judgement: **low risk** Support for judgement: all outcome data available	Authors’ judgement: **low risk** Support for judgement: outcome assessors blinded	Authors’ judgement: **low risk** Support for judgement: reported outcome data unlikely to have been selected	Authors’ judgement: **high risk**
2	Alswafeeri et al. 2015 [17]	Authors’ judgement: **some concerns** Support for judgement: randomization process adequate but allocation concealment process not described	Authors’ judgement: s**ome concerns** Support for judgement: carers and trial personnel aware of participants’ assigned intervention	Authors’ judgement: **low risk** Support for judgement: all outcome data available	Authors’ judgement: **high risk** Support for judgement: outcome assessors not blinded	Authors’ judgement: **low risk** Support for judgement: reported outcome data unlikely to have been selected	Authors’ judgement: **high risk**
3	Wahab et al. 2014 [18]	Authors’ judgement: **high risk** Support for judgement: quasi-randomization process and allocation concealment process not described	Authors' judgement: **some concerns** Support for judgement: carers and trial personnel aware of participants’ assigned intervention	Authors’ judgement: **low risk** Support for judgement: all outcome data available	Authors’ judgement: **high risk** Support for judgement: outcome assessors not blinded	Authors’ judgement: **low risk** Support for judgement: reported outcome data unlikely to have been selected	Authors’ judgement: **high risk**
4	Barbieri et al. 2013 [19]	Authors’ judgement: **high risk** Support for judgement: no details provided about randomization and allocation concealment processes	Authors’ judgement: **some concerns** Support for judgement: carers and trial personnel aware of participants’ assigned intervention	Authors’ judgement: **low risk** Support for judgement: all outcome data available	Authors’ judgement: **high risk** Support for judgement: outcome assessors not blinded	Authors’ judgement: **low risk** Support for judgement: reported outcome data unlikely to have been selected	Authors’ judgement: **high risk**
5	Kalha et al. 2010 [20]	Authors’ judgement: **high risk** Support for judgement: no details provided about randomization and allocation concealment processes	Authors’ judgement: **some concerns** Support for judgement: carers and trial personnel aware of participants’ assigned intervention	Authors’ judgement: **low risk** Support for judgement: all outcome data available	Authors’ judgement: **high risk** Support for judgement: outcome assessors not blinded	Authors’ judgement: **low risk** Support for judgement: reported outcome data unlikely to have been selected	Authors’ judgement: **high risk**

#### Non-RCTs

Nine non-RCTs were identified. None was rated at low risk of bias. Five of the included studies were rated at moderate risk of bias [21, 22, 24, 27, 28]. Two studies were rated at serious risk of bias [23, 29] and another two at critical risk of bias [25, 26]. Detailed assessment of their risk of bias is depicted in Table 3.

**Table 3:** Risk of bias of included non-randomized studies

Author/year of publication	Bias due to confounding	Bias in selection of participants into the study	Bias in classification of interventions	Bias due to deviations from intended interventions	Bias due to missing data	Bias in measurement of outcomes	Bias in selection of the reported result	Overall
Bitra et al. 2017 [21]	Low risk	Low risk	Low risk	Low risk	Low risk	Moderate risk	Low risk	Moderate risk
Smuthkochorn et al. 2017 [22]	Low risk	Low risk	Low risk	Low risk	Low risk	Moderate risk	Low risk	Moderate risk
Yang et al. 2014 [23]	Serious risk	Low risk	Low risk	Low risk	Low risk	Moderate risk	Low risk	Serious risk
Alfaqeeh et al. 2011 [24]	Low risk	Low risk	Low risk	Low risk	Low risk	Moderate risk	Low risk	Moderate risk
Batra et al. 2006 [25]	Critical risk	Low risk	Low risk	Low risk	Low risk	Moderate risk	Low risk	Critical risk
Isik et al. 2005 [26]	Critical risk	Low risk	Low risk	Low risk	Low risk	Moderate risk	Low risk	Critical risk
Perinetti et al. 2004 [27]	Low risk	Low risk	Low risk	Low risk	Low risk	Moderate risk	Low risk	Moderate risk
Perinetti et al. 2002 [28]	Low risk	Low risk	Low risk	Low risk	Low risk	Moderate risk	Low risk	Moderate risk
Griffiths et al. 1998 [29]	Serious risk	Low risk	Low risk	Low risk	Low risk	Moderate risk	Low risk	Serious risk

#### Cross-sectional studies

One cross-sectional study was rated with 4 stars (moderate quality), according to the Newcastle-Ottawa Quality assessment scale [30] (Table 4).

**Table 4:** Quality assessment of included cross-sectional study

Author/year of publication	Selection			Comparability	Outcome		Overall
	Representativeness of the sample	Nonrespondents	Ascertainment of the exposure		Assessment of the outcome	Statistical test	
Insoft el al. 1996 [30]	1 star	-	1 star	-	2 stars	-	4/7 stars

### Quantitative synthesis of the included studies

Due to the great heterogeneity between the interventions, the number of participants, the biomarkers assessed, and the follow-up period among studies, a meta-analysis was not feasible. The bias within studies and the fact that design of included studies has been diverse, have precluded, thus, a valid interpretation of the results through pooled estimates. Only qualitative assessment as a narrative review has been performed and reported (Table 5). The overall quality of evidence according to GRADE system was rated as low for NTX and TRAP or very low for the OPN, ALP, and OC (Table 6).

**Table 5:** Outcomes of included studies

Authors Study design	Changes in GCF/time point biomarkers	Measurement unit	Biological consequence	Clinical significance
Castroflorio et al. 2017 [16] RCT/ Split mouth	OPN test sites: T0: 28.1 ± 15.5 T1: 35.6 ± 19.9 T2: 35.2 ± 18.5 T3: 46.0 ± 22.7 Control sites: T0: 31.2 ± 15.7 T1: 26.2 ± 13.6 T2: 32.9 ± 14.5 T3: 31.3 ± 14.7	ng/μl	Invisalign aligners release an initial force of about 1 N on distalizing a maxillary molar. This force delivery produces an increased concentration of OPN at tension sites.	Not reported
Al Swafeeri et al. 2015 [17] RCT/ Split mouth	Constant force group Canine A0 9.16 ± 5.22 A1 12.10 ± 7.86 A2 23.19 ± 9.83 A3 21.73 ± 12.16 Molar B0 7.19 ± 6.26 B1 9.32 ± 6.57 B2 21.23 ± 9.70 B3 13.95 ± 8.05 Gradually increasing force group Canine C0 10.66 ± 6.99 C1 19.50 ± 12.41 C2 35.73 ± 10.10 C3 20.22 ± 12.88 Molar D0 5.70 ± 6.39 D1 11.42 ± 5.05 D2 18.74 ± 5.48 D3 27.03 ± 9.32	IU/μl	The use of a gradually increasing orthodontic force could induce an increase in osteoblastic activity during initial stage of orthodontic tooth movement compared with that induced by a relatively constant orthodontic force.	Gradually increasing force systems could be recommended for clinical use in orthodontics.
Wahab et al. 2014 [18] RCT/ Split mouth	The LDH activity for 100 g of force exhibited significant differences at weeks 2 and 3, while the activities of AST and TRAP were significantly different from control values at week 5. For 150 g of force, there were significant differences in the LDH activities at weeks 3, 4, and 5 but no significant differences in the TRAP activities.	U/mg	The activity of LDH in the GCF increased significantly at 2 and 3 weeks for 100 g of force and at 1, 2, 3 weeks for 150 g of force. These findings showed that inflammation occurred earlier when 150 g of force was applied, which might induce a painful sensation that starts earlier and lasts longer. The 150-g force is a heavy force, and it produced significant AST-specific activity or necrosis earlier than the application of 100-g force. TRAP activity shows no differences for 150g of force throughout the treatment, this finding indicates that heavy force results in undermining resorption	The movements of the canines showed no significant differences between 100 and 150 g of force throughout the 5 weeks of treatment. LDH, TRAP, and AST from the GCF may be used as biomarkers for monitoring orthodontic tooth movement
Barbieri et al. 2013 [19] RCT/ Split mouth	At the control sites there were no differences between the values recorded for buccal/palatal sites or between values recorded at different visits. In contrast, the concentration of OPG significantly decreased at the compression site by 24 h and the amount, and concentration of RANK differed significantly between control, compression, and tension sites after 7 days. A significant increase in absolute TGF-β1 levels was also detected at the compression site versus the control and tension sides after 7 days.	pg/ml or pg	Both increased expression of bone resorptive mediators (RANK, TGFβ1) and decreased expression of a bone-forming mediator (OPG) on the compression side were detected.	Bone metabolism is affected by application of force to the teeth by elastic separators.
Kalha et al. 2010 [20] RCT	There was a 200% increase in the alkaline phosphatase level between days 21 and 28 in the active tie-back group at all sites, while that in the retraction screw group was more than 260%.	IU/l	Alkaline phosphatase levels increased more in the Hycon-screw group between 14 and 28 days. That can be explained by the fact that elastomeric modules generally lose 50–70% of their initial force after 3 weeks of loading.	Sequential repetitive loading of the periodontal ligament with small and controlled activations is effective for space closure as indicated by a significantly higher increase in the GCF alkaline phosphatase level if a retraction screw is used instead of active tie-backs.
Bitra et al. 2017 [21] Prospective	OPN: Baseline: Premenopausal 241.52 pg/μl and postmenopausal 317.15 pg/μl 24 h after: Premenopausal 540.97 pg/μl and postmenopausal 492.73 pg/μl	pg/μl	No difference is observed in GCF levels of OPN while comparing the mean premenopausal and postmenopausal women.	Orthodontic treatment appears to be equally safer for both premenopausal and postmenopausal subjects.
Smuthkochorn et al. 2017 [22] Prospective	OPN Premenopausal T0 238.92 ± 66.88 T1 531.72 ± 465.98 Postmenopausal T0 323.26 ± 157.27 T1 489.61 ± 280.38	pg/μl	There are OPN baseline differences in GCF bone turnover markers in premenopausal vs. postmenopausal groups.	Reactions to orthodontic activation are not significantly different between the groups.
Yang et al. 2014 [23] Prospective	The OCN levels were significantly higher in the ovulation period group than in the menstrual period group (*P* < 0.05).	pg/nl	The OCN levels affected from the menstrual period	Exerted force on teeth during the menstrual period may promote rapid tooth movement.
Alfaqeeh et al. 2011 [24] Prospective	At the experiment sites, the GCF NTX level steadily increased from day 7 to day 21. The control side did not show any statistically significant variation throughout the observation period. The OC levels in the GCF showed no difference at the control site. At the experimental site where pressure was applied, the levels of OC showed higher values on day 7 and onward.	nmol of bone collagen equivalents per litre (nmol BCE/L)	Statistically significant changes in NTX and OC levels on days 7, 14, and 21 when we compared the experimental and control sides. The peak in all activity of the variables occurred on day 14 after retraction.	The GCF NTX and OC markers showed statistically significant increases in the levels on days 14 and 21 at the side that had orthodontic tooth movement.
Batra et al. 2006 [25] Prospective	Significant changes on ALP activity on 7, 14, and 21 days at mesial & distal sides compared with experimental and control teeth	IU/l	The peak in enzyme activity occurred on the 14th day of initiation of retraction followed by a significant fall in activity.	ALP activity could possibly be a biological indicator of the activity in the periodontium and orthodontic tooth movement.
Isik et al. 2005 [26] Prospective	OC: Initial 500.22 ± 360.67 1 h 500.22 ± 360.67 1 day 451.86 ± 330.07 7 days 505.85 ± 236.15 22 days 326.98 ± 240.58 28 days 309.22 ± 173.85 BALP: Initial 102.08 ± 63.62 1 h 91.82 ± 65.07 1 day 57.39 ± 33.48 7 days 75.14 ± 49.56 22 days 44.60 ± 24.17 28 days 52.35 ± 27.10 DPD: Initial 1.54 ± 0.57 1 h 1.16 ± 0.66 1 day 0.95 ± 0.56 7 days 0.85 ± 0.50 22 days 0.52 ± 0.12 28 days 0.75 ± 0.48 NTX: Not detectable	pmol/mg	DPD, OC, and BALP values decrease with force application.	The applied forces may have caused the hyalinization process and decrease of bone turnover.
Perinetti et al. 2004 [27] Prospective	ALP: Test tooth: Mesial • T0 60 ± 36 • 28 days 159 ± 83 Distal • T0 52 ± 18 • 28 days 102 ± 43 Contralateral tooth: Mesial • T0 59 ± 33 • 28 days 80 ± 31 Distal • T0 61 ± 23 • 28 days 81 ± 26 Antagonist tooth: Mesial • T0 46 ± 26 • 28 days 47 ± 24 Distal • T0 43 ± 19 • 28 days 44 ± 24	mU/sample	GCF ALP is sensitive in distinguishing between tension and compression sites on day 28.	Factors as clinical condition changes may affect the enzymatic activities. GCF ALP should be considered as a reliable biomarker of tissue responses to orthodontic treatment only when oral hygiene is kept under control.
Perinetti et al. 2002 [28] Prospective	Distalized molar: Baseline 60 ± 33 1 h 79 ± 53 7 days 138 ± 95 14 days 160 ± 82 21 days 147 ± 101 28 days 153 ± 84 Contralateral molar: Baseline 43 ± 25 1 h 69 ± 52 7 days 74 ± 44 14 days 90 ± 52 21 days 73 ± 43 28 days 75 ± 38 Antagonist molar: Baseline 50 ± 24 1 h 46 ± 30 7 days 45 ± 18 14 days 50 ± 15 21 days 37 ± 21 28 days 46 ± 23	mU/sample	High levels of ALP activity have been described after 7 days when bone deposition begins, greater in dental sites of tension than in sites of compression.	Increased ALP activity in teeth-bearing orthodontic appliances might be due to gingival inflammation independently of clinically detectable movement.
Griffiths et al. 1998 [29] Prospective	PYD, DPD not detected OC showed variation between subjects and within subject at different stages	pg/ml or pg	OC has been shown to be present in the GCF of adolescents, but there was a wide variation between subjects in the amount and concentration.	GCF volume is the most sensitive indicator of gingival inflammation.
Insoft et al. 1996 [30] Cross-sectional	Acid phosphatase activities were constantly higher in all groups. There was a substantial peak in acid phosphatase at month 11, followed by a smaller second peak at month 16, and an even smaller at month 19. ALP demonstrated a large first peak at month 15 and a second smaller at month 17.5.	sigma units/min	Changes in GCF phosphatase activities during orthodontic tooth movement may reflect bone remodeling.	ALP in GCF is partly influenced by gingival inflammation.

*GCF* gingival crevicular fluid, *BTMs* bone turnover markers, *OTM* orthodontic tooth movement, *BALP* bone alcaline phosphatase, *ALP* alcaline phosphatase, *OC* osteocalcin, *LD* lactate dehydrogenase, *DPD* deoxypyridinoline, *PYD* pyridinoline, *NTX* N-terminal telopeptide, *OPN* osteopontin, *TRAP* tartrate-resistant acid phosphatase, *PDL* periodontal ligament, *PINP* N-terminal collagen type I extension pro-peptide, *CTX* C-terminal cross-linking telopeptide of type I collagen, *RCTs* randomized clinical trials, *IL-1β* interleukin 1b, *TNF* tumor necrosis factor a, *RANKL* receptor activator of nuclear factor-κB ligand

**Table 6:** Summary of findings according to the GRADE approach. Population: orthodontic patients of any age and sex. Intervention: any type of removable or fixed orthodontic appliance resulting in OTM. Comparisons: any control group was accepted, i.e. untreated group, contralateral sides in split mouth design, control group with different type of orthodontic activations (i.e. force applied and constant or increasing forces)

Outcomes	Quality of the evidence (GRADE)	No. of participants (studies)	Comments
Alcaline phosphatase (ALP)	⊕OOO Very low ^**a**^ Due to inconsistency and indirectness	118 (6)	
Osteopontin (OPN)	⊕OOO Very low ^**b**^ Due to indirectness	20 (2)	2 studies were excluded because they had different comparison groups
Osteocalcin (OC)	⊕OOO Very low ^**c**^ Due to indirectness	32 (2)	2 studies excluded due to the lack of comparison group
N-telopeptides (NTX)	⊕⊕OO Low ^**d**^	20 (1)	One study was excluded due to the lack of comparison group
Tartate-resistant acid phosphatase (TRAP)	⊕OOO Very low ^**e**^	19 (1)	

^a^Downgraded by two levels for bias due to high risk of bias for both included randomized studies and due to the inclusion of non-randomized studies with critical/serious risk of bias

^b^Downgraded by two levels for bias due to high risk of bias for both included randomized studies

^c^Downgraded by two levels for bias due to the inclusion of non-randomized studies with moderate/serious risk of bias

^d^Downgraded because this is a non-randomized study

^e^Downgraded by two levels for bias due to high risk of bias for both included randomized studies

Deoxypyridinol (DPD), bone alkaline phosphatase (BALP), and pyridinoline (PYD) are not included in the table since there is no comparison group in the included studies

### Qualitative synthesis of the included studies

#### Type of orthodontic intervention

Most of the studies evaluated the GCF of an upper canine prior, during, and after distalization. The other maxillary canine served as control [17, 18, 20, 23–25, 27, 29]. Several studies detected the biomarkers in various teeth under orthodontic treatment with fixed appliances [21, 22, 26, 28] or after the placement of separators [19]. One study evaluated the GCF of patients with aligners [16]. A headgear and a Bionator were the intervention in one study [30].

#### Biomarkers assessed

The following biomarkers for bone formation were assessed: bone alcaline phosphatase (BALP), alcaline phosphatase (ALP), and osteocalcin (OC).

The following biomarkers for bone resorption were assessed: deoxypyridinoline (DPD) and pyridinoline (PYD), N-terminal telopeptide (NTX), osteopontin (OPN), and tartrate-resistant acid phosphatase (TRAP). The follow-up period ranged mainly from baseline to 45 days. One study had an expanded follow-up period of up to 16 months [29] (Table 1).

##### Biomarkers of bone formation

**Bone alcaline phosphatase (BALP) and alcaline phosphatase (ALP)**

ΒALP was examined in one study [26]. Although BALP values showed a descending character after activation visits, no statistically significant difference was reported overall. ALP was examined in 7 studies [17, 18, 20, 25, 27, 28, 30]. One study found no statistically significant differences in ALP levels compared with baseline [18]. Alswafeeri et al. compared two groups during maxillary canine distalization with constant continuous vs. gradually increasing retraction forces. They found a specific pattern of the ALP activity in the constant force group [17]. This pattern included an initial rise from baseline to the 1st week, then a peak in the 2nd week. This peak was followed by a reduction in enzymatic activity in the 3rd week. Overall increases in enzymatic activity in the constant force group were lower than in the gradually increasing force group. Besides, the use of a gradually increasing orthodontic force could induce an increase in osteoblastic activity during the initial stage of OTM compared with that induced by a relatively constant orthodontic force [17]. Kalha et al. compared two groups of patients during space closure with Hycon-screw vs. active-tie backs. Increased levels were found in both groups; however, ALP increased more in the Hycon-screw group, due to the rapid initial force decay of the elastomeric modules. For the same reason, they concluded that the sequential repetitive loading of the periodontal ligament with the small and controlled activations of the screw was more effective for space closure [20]. Batra et al. detected significant differences in ALP on days 7, 14, and 21. On days 7 and 14, ALP was increased whereas on day 21 declined [25]. In the study of Perinetti et al., ALP levels during molar distalization were significantly higher from day 7 until the end of the treatment. The ALP levels were significantly higher in contralateral teeth, too [28]. In another study of Perinetti et al., the GCF ALP activity significantly increased over time in both the mesial and the distal sites of the experimental teeth and the mesial sites of the contralateral. In the distal sites of contralateral teeth, there was an ALP activity increase, although not significant [27]. Finally, in the antagonist teeth, this enzymatic activity was stable throughout the study, without any statistically significant changes. On day 28, enzymatic activity was significantly greater in the experimental teeth, as compared with the contralateral teeth [27]. Both studies of Perinetti et al. revealed that ALP levels were higher at tension sites than in sites of compression. Insoft et al. stated that ALP levels peaked between the 1st and 3rd week after initiation of tooth movement. Additionally, ALP increased with inflammation in treated groups [30].

**Osteocalcin (OC)**

OC was assessed in 4 out of 55 studies [23, 24, 26, 29]. During canine retraction for a follow-up period of 28 days, Alfaqeeh et al. found the peak levels of OC on days 14 and 21 [24]. Yang et al. found that OC levels in teeth under orthodontic movement were significantly higher in women in the ovulation period than in the menstrual period [23]. Isik et al. observed a descending character of OC levels, with the exception of a slight rise on the 7th day. The aforementioned changes were not statistically significant [26]. Griffiths et al. evaluated OC levels prior, during and after canine retraction and identified a higher concentration of OCN after fixed appliance fit, but no specific conclusion could be drawn due to the great variety between the findings of the sample [29].

##### Biomarkers of bone resorption

**Deoxypyridinoline (DPD) and pyridinoline (PYD)**

DPD was evaluated in two studies [26, 29]. According to Isik et al., DPD values showed a decreasing trend during tooth intrusion from 1 h to 28 days. That decrease was statistically significant at 22 and 28 days after force application [26]. On the other hand, Griffiths et al. could not detect DPD in GCF prior, during, or after canine retraction [29].

**N-terminal telopeptide (NTX)**

NTX was investigated in 2 out of 5 studies [24, 26]. Alfaqeeh et al. demonstrated that NTX levels increased steadily during canine retraction. Significant differences between experimental and control sites were observed on day 14 and 21 after the initiation of the treatment with maximum NTX levels at the end of the experiment, on the 21st day [24].

However, in the Isik et al. study, NTX values were found to be below the detection limit with a few readings which showed large variations between subjects and stages of tooth movement [26].

**Osteopontin (OPN)**

OPN was investigated in 4 studies [16, 19, 21, 22]. Castroflorio et al. reported that the kinetics of OPN was characterized by a significant increase at the tension sites of the test teeth after 3 weeks from the application of orthodontic force [16]. Barbieri et al. found that the concentration of OPN significantly decreased at the compression site 24 h after initiation of tooth movement with elastic separators [19]. The other two studies came to the same conclusion (i.e. that there is no difference in the response to orthodontic activation between premenopausal and postmenopausal, as long as OPN is concerned) [21, 22].

**Tartrate-resistant acid phosphatase (TRAP)**

TRAP was detected only in one study [18]. In the group of 100-g force, the TRAP levels were significantly elevated in the 5th week after force application compared with baseline. In contrast, the levels of TRAP in the group of 150-g force remained the same during the observational period. This finding indicated that light force has the ability to evoke frontal resorption of the bone [18].

## Discussion

The aim of the present systematic review was to provide an updated summary of the available evidence regarding the collection of biomarkers in GCF, so as to guide and facilitate future research projects. The included studies demonstrated high heterogeneity, regarding methodological, clinical, and statistical issues. Clinical heterogeneity among studies included considerable variations in participants (sample size, age, and sex) as well as in interventions (follow-up, orthodontic type of intervention), whereas the diversity in the measurement units of the biomarkers indicated considerable methodological heterogeneity. The aforementioned forms of heterogeneity precluded the possibility for a valid meta-analysis.

The inclusion criteria for most of the studies were good general health, no history of antibiotic therapy during the previous months or anti-inflammatory drug use within 1 month before GCF collection in periodontally healthy nonsmokers. One week to 1 month prior to GCF collection, the participants underwent a session of professional supra- and sub-gingival scaling and also received repeated oral hygiene instructions [19, 27].

Most studies evaluated the biomarkers in GCF samples before, during, and after canine distalization in cases of first premolar extractions [17, 18, 20, 23–25, 27, 29] or in a tooth that received active force during fixed appliance activation [21, 22, 26, 28]. It should be pointed out that only two studies investigated the role of the force magnitude [17, 18].

There was no agreement between the studies regarding GCF sample collection and management. Several differences were identified during the following stages:

- Isolation of the sites of GCF collection (most often with cotton rolls),
- Method of GCF collection (paper strips, micropipettes),
- Depth insertion of paper strips,
- One single or repeated measurements,
- Time that paper strips remain inside the gingival sulcus (e.g. 30 or 60 s),
- Time slot of the day for the collection,
- Incubation solution which was used for the GCF sample (e.g. phosphate-buffered saline),
- Biochemical assay used for the analysis of biomarkers (e.g. Elisa, Western blot).

The fluctuation of the levels of biomarkers in GCF is suggestive of underlying intricate biological remodeling processes in bone and periodontal tissues related to OTM [1]. Mechanical stimulus causes an inflammatory reaction within the periodontal tissues, which in turn may trigger the biological processes associated with bone remodeling [1]. There is a systematic review reported that mechanical stress induces acute inflammatory changes that alter the microvascular environment and provoke local release of mediators interleukin 1b (IL-1β), tumor necrosis factor a (TNF-α), as well as expression of chemokines that ultimately promote leukocyte adhesion and migration [31]. Whether this reaction is inflammatory or not is a subject for debate. The same research team, more recently, conducted another systematic review in their attempt to establish associations between enzymes in GCF, force magnitude, and site of application [32]. Concerning ALP and TRAP, markers, assessed also in our study, reported that ALP was increased in the tension site after 7 days, while TRAP showed a later peak, namely after 4–5 weeks, in the compression site. Both TRAP and ALP levels were greater in the 150-g force than in the 100-g force [32]. This conclusion comes in contrast with the findings of our review, according to which TRAP levels remained stable after the 150-g force application. Meikle (2006) stated that tooth movement met only the last of the four classical criteria for inflammation (redness, heat, swelling, and pain), suggesting instead that the process should be best regarded as an exaggerated form of normal physiological turnover combined with tissue repair [33].

The increase of these pro-inflammatory cytokines results in chronic leukocyte recruitment and tissue destruction and seems to play a crucial role in periodontal remodeling during tooth movement, preventing pathological destruction of the bone and PDL [34]. The amount, the rate, and the function of the released biomarkers not only reflect the activity of individual cells but also indicate the metabolic activity in the involved tissues or organs [35].

In the past two decades, there has been significant interest in the development of noninvasive oral and systemic diagnostic biomarkers by large-scale protein analysis. Whole saliva, parotid secretions, and GCF samples have been collected for diagnostic biomarker discovery. The notion to use GCF as a source of diagnostic biomarker is not uncommon; however, the possibility of using a panel of independent disease-related proteins has recently emerged. In this respect, the ability to highlight a large number of proteins with local tissue/cell specificity and to define their relative levels in health versus disease have become of major interest.

The use of BTMs for the monitoring of treatment requires a baseline assessment with a repeat measurement at some defined point during orthodontic treatment. In order to do this effectively, it is important to assess the expected level of alteration. Thus, it is important to monitor treatment effect in the individual, the imprecision of the measurement, as well as the intra-individual variability which may be influenced by factors such as timetable of sampling, fasting status, adherence to instructions, etc..

### Strengths & limitations

Some limitations do exist in the present review. Ideally, only randomized trials with control groups would be included in this review. However, due to the scarcity of available studies in the field, non-randomized designs were also considered for eligibility. Inter-rater reliability during data extraction was not tested; nevertheless, this has probably low impact as consensus was reached with the last author, when needed. The lack of blinding and generally the methodological heterogeneity in the included studies may have also introduced uncertainty in the results. However, the main strength of this review is that it gathers information about GCF collection and BTM values so that the future studies can be conducted under standardized conditions, with the sole purpose of using BTM in regulating orthodontic tooth movement.

### Implications for research

In summary, the available studies relating BTM changes after an orthodontic stimulus are promising. Based on the results of this literature review, several guidelines for standardization may be suggested:

- The diversity in expressing the released quantities and the use of different units hindered this review. To allow unequivocal interpretation and comparison between different studies, it is recommended to express quantitative release data in standardized units. The use of internationally agreed decision limits and target values for these markers requires that measurements are universally comparable. Standardization and establishment of a reference system for the BTMs is the route to achieve this [36, 37].
- The limits for detection/quantification of each analyzed eluate are essential for the interpretation of the results, and should therefore always be mentioned. Compounds that could not be detected, may still have been released, but in concentrations below the detection limit. It would thus not be correct to assume that they are not released in the GCF.
- Contamination may lead to false-positive detection of compounds, and great care should be taken to avoid any contamination. All studies should report if the necessary measures were taken in order for contamination from saliva to be avoided.

### Implications for clinical practice

- Too often, the materials and methods failed to mention necessary information about the GCF collection procedure. Information such as the volume of incubation solution, the percentage of solvent in case of dilutions, the pH of the solution, and the brand of paper strips should be always stated.
- As BTMs may show significant responses to the orthodontic treatment, their response to treatment may allow the best choice of a possible future chemical or pharmacological agent. They may also help with the proof of principle and help establish the mechanism of action. This could potentially alter the actual orthodontic treatment modalities.

## Conclusions

Current evidence continues to support the potential for BTMs to provide clinically useful information particularly for adjusting or standardizing the orthodontic stimulus, and in the future for modulating the orthodontic tooth movement. The present systematic review has retrieved studies of high, overall, risk of bias, and has unveiled a substantial clinical and methodological heterogeneity among included studies. Further data of the relationships between the clinical assays and the physiological or pre-analytical factors contributing to variability in BTMs’ concentrations are required.

### Other information

The review protocol was specified in advance and registered at PROSPERO (International Prospective Register of Systematic Reviews), No. CRD42020212056.

## Supplementary Information

**Additional file 1.** PRISMA 2020 for Abstracts Checklist.

**Additional file 2.** PRISMA 2020 Checklist.

**Additional file 3 **Search strategy, MEDLINE/*PubMed, assessed as up to date: 01.09.2020.*

**Additional file 4.** LILACS, Cochrane Library, MEDLINE, and Embase search strategies.

**Additional file 5.** List of excluded studies.

## Data Availability

The data underlying this article are available in the article and in its online supplementary material.

## Abbreviations

GCF: Gingival crevicular fluid
BTMs: Bone turnover markers
OTM: Orthodontic tooth movement
BALP: Bone alcaline phosphatase
ALP: Alcaline phosphatase
OC: Osteocalcin
DPD: Deoxypyridinoline
PYD: Pyridinoline
NTX: N-terminal telopeptide
OPN: Osteopontin
TRAP: Tartrate-resistant acid phosphatase
PDL: Periodontal ligament
PINP: N-terminal collagen type I extension pro-peptide
CTX: C-terminal cross-linking telopeptide of type I collagen
PRISMA: Preferred Reporting Items for Systematic Reviews and Meta-Analyses
RCTs: Randomized clinical trials
IL-1 β: Interleukin 1b
TNF- α: Tumor necrosis factor a
