# Supplementary material for: Fluctuation of bone turnover markers’ levels in samples of gingival crevicular fluid after orthodontic stimulus: a systematic review
Source: Syst Rev. 2022 Jan 4;11:3. doi: 10.1186/s13643-021-01860-w (PMC8725272; doi:10.1186/s13643-021-01860-w)
Supplement: Supplementary file 3 — Additional file 3 Search strategy, MEDLINE/PubMed, assessed as up to date: 01.09.2020. [file 13643_2021_1860_MOESM3_ESM.pdf]

Search strategy, Medline/*Pubmed , assessed as up to date:* **01.10.2020**

#1 periodontal biomarker*	2417
#2 periodontal biomarker* AND orthodont*	136
#3 biomarker* AND orthodont*	501
#4 biomarker*[tiab] AND orthodont*	233
#5 biomarker*[tiab] AND orthodont*[tiab]	93
#6 ((periodontal) AND biological marker) AND orthodont*	184
#7 GCF AND orthodontic	218
#8 GCF AND biomarkers	492
#9 GCF biomarkers AND orthodont*	60
#10 GCF AND orthodont*	214
#11 gingival crevicular fluid	4107
#12 gingival crevicular fluid AND orthodont*	307
#13 gingival crevicular fluid AND orthodont* [tiab]	269
#14 gingival crevicular fluid biomarkers	708
#15 gingival crevicular fluid biomarkers AND orthodont*	75
#16 gingival crevicular fluid[MeSH Terms]	3295
#17 (gingival crevicular fluid[MeSH Terms]) AND orthodont*[Title/Abstract]	211
#18 Bone turnover[tiab] AND orthodont*[tiab]	78
#19 "bone"[Title/Abstract] AND ("turnover"[Title/Abstract] OR "remodel*"[Title/Abstract]) AND "orthodont*"[Title/Abstract]	747
#20 "bone"[Title/Abstract] AND ("turnover"[Title/Abstract] OR "remodel*"[Title/Abstract]) AND "orthodont*"[Title/Abstract] AND ("biomarker s"[All Fields] OR "biomarkers"[MeSH Terms] OR "biomarkers"[All Fields] OR "biomarker"[All Fields])	59
