# Supplementary material for: Fluctuation of bone turnover markers’ levels in samples of gingival crevicular fluid after orthodontic stimulus: a systematic review
Source: Syst Rev. 2022 Jan 4;11:3. doi: 10.1186/s13643-021-01860-w (PMC8725272; doi:10.1186/s13643-021-01860-w)
Supplement: Supplementary file 4 — Additional file 4. LILACS, Cochrane Library, MEDLINE, and Embase search strategies. [file 13643_2021_1860_MOESM4_ESM.pdf]

LILACS search strategy

1. biomarker [Words] and orthodontic [Words] 5
2. GCF [Words] and orthodontic [Words] 12
3. gingival crevicular fluid [Words] and orthodontic [Words] 9
4. bone turnover [Words] and orthodontic [Words] 40
5. periodontal biomarker [Words] and orthodontic [Words] 0

Cochrane Library search

#1
(biomarker):ti,ab,kw AND (orthodont*):ti,ab,kw
(biomarker):ti,ab,kw AND (orthodont*):ti,ab,kw
(Word variations have been searched)
29

#2
(GCF):ti,ab,kw AND (orthodont*):ti,ab,kw
(GCF):ti,ab,kw AND (orthodont*):ti,ab,kw
(Word variations have been searched)
69

#3
("bone turnover marker"):ti,ab,kw AND (orthodont*):ti,ab,kw
("bone turnover marker"):ti,ab,kw AND (orthodont*):ti,ab,kw
(Word variations have been searched)
1

#4
("gingival crevicular fluid"):ti,ab,kw AND (orthodont*):ti,ab,kw
("gingival crevicular fluid"):ti,ab,kw AND (orthodont*):ti,ab,kw
(Word variations have been searched)
90

Medline (via Ovid)

1. (biomarker and orthodont*).ab. 32
2. (GCF and orthodont*).ab. 194
3. (gingival crevicular fluid and orthodont*).ab. 231
4. (bone turnover and orthodont*).ab. 76
5. Gingival crevicular fluid.sh. and orthodont*.ab. 208
6. orthodont*.sh. and GCF.ab. 94

Embase (via Ovid)

1. (biomarker and orthodont*).ab. 38
2. (GCF and orthodont*).ab. 197
3. (gingival crevicular fluid and orthodont*).ab. 240
4. (bone turnover and orthodont*).ab. 81
5. gingival crevicular fluid.af. and orthodont*.ab. 249
