# Supplementary material for: Fluctuation of bone turnover markers’ levels in samples of gingival crevicular fluid after orthodontic stimulus: a systematic review
Source: Syst Rev. 2022 Jan 4;11:3. doi: 10.1186/s13643-021-01860-w (PMC8725272; doi:10.1186/s13643-021-01860-w)
Supplement: Supplementary file 5 — Additional file 5. List of excluded studies. [file 13643_2021_1860_MOESM5_ESM.pdf]

**Appendix 3- List of excluded studies**


1. Alarcón JA, Linde D, Barbieri G, Solano P, Caba O, Rios-Lugo MJ, Sanz M, Martin C. Calcitonin gingival crevicular fluid levels and pain discomfort during early orthodontic tooth movement in young patients. Arch Oral Biol. 2013 Jun;58(6):590-5. doi: 10.1016/j.archoralbio.2012.10.002. Epub 2012 Oct 26. PMID: 23107048.
2. Andrade I Jr, Taddei SR, Garlet GP, Garlet TP, Teixeira AL, Silva TA, Teixeira MM. CCR5 down-regulates osteoclast function in orthodontic tooth movement. J Dent Res. 2009 Nov;88(11):1037-41. doi: 10.1177/0022034509346230. PMID: 19828893.
3. Apajalahti S, Sorsa T, Railavo S, Ingman T. The in vivo levels of matrix metalloproteinase-1 and -8 in gingival crevicular fluid during initial orthodontic tooth movement. J Dent Res. 2003 Dec;82(12):1018-22. doi: 10.1177/154405910308201216. PMID: 14630906.
4. Atuğ Özcan SS, Ceylan I, Ozcan E, Kurt N, Dağsuyu IM, Canakçi CF. Evaluation of oxidative stress biomarkers in patients with fixed orthodontic appliances. Dis Markers. 2014;2014:597892. doi: 10.1155/2014/597892. Epub 2014 Apr 10. PMID: 24864131; PMCID: PMC4016902.
5. Baldwin PD, Pender N, Last KS. Effects on tooth movement of force delivery from nickel-titanium archwires. Eur J Orthod. 1999 Oct;21(5):481-9. doi: 10.1093/ejo/21.5.481. PMID: 10565088.
6. Başaran G, Ozer T, Kaya FA, Kaplan A, Hamamci O. Interleukine-1beta and tumor necrosis factor-alpha levels in the human gingival sulcus during orthodontic treatment. Angle Orthod. 2006 Sep;76(5):830-6. doi: 10.1043/0003-3219(2006)076[0830:IATNFL]2.0.CO;2. PMID: 17029518.
7. Bildt MM, Bloemen M, Kuijpers-Jagtman AM, Von den Hoff JW. Matrix metalloproteinases and tissue inhibitors of metalloproteinases in gingival crevicular fluid during orthodontic tooth movement. Eur J Orthod. 2009 Oct;31(5):529-35. doi: 10.1093/ejo/cjn127. Epub 2009 Mar 18. PMID: 19299245.
8. Bolamperti L, Montanari P, Levrini L, Macchi A, Tagliabue A, Caprioglio A. Tissue response during self-ligating treatment. Prog Orthod. 2012 Sep;13(2):109-16. doi: 10.1016/j.pio.2011.11.001. Epub 2012 Feb 14. PMID: 23021113.
9. Burke JC, Evans CA, Crosby TR, Mednieks MI. Expression of secretory proteins in oral fluid after orthodontic tooth movement. Am J Orthod Dentofacial Orthop. 2002 Mar;121(3):310-5. doi: 10.1067/mod.2002.121011. PMID: 11941346.
10. Canavarro C, Teles RP, Capelli Júnior J. Matrix metalloproteinases -1, -2, -3, -7, -8, -12, and -13 in gingival crevicular fluid during orthodontic tooth movement: a longitudinal randomized split-mouth study. Eur J Orthod. 2013 Oct;35(5):652-8. doi: 10.1093/ejo/cjs053. Epub 2012 Sep 17. PMID: 22989715.
11. Cantarella G, Cantarella R, Caltabiano M, Risuglia N, Bernardini R, Leonardi R. Levels of matrix metalloproteinases 1 and 2 in human gingival crevicular fluid during initial tooth movement. Am J Orthod Dentofacial Orthop. 2006 Nov;130(5):568.e11-6. doi: 10.1016/j.ajodo.2006.04.020. PMID: 17110252.
12. Capelli J Jr, Kantarci A, Haffajee A, Teles RP, Fidel R Jr, Figueredo CM. Matrix metalloproteinases and chemokines in the gingival crevicular fluid during orthodontic tooth movement. Eur J Orthod. 2011 Dec;33(6):705-11. doi: 10.1093/ejo/cjq148. Epub 2011 Mar 9. PMID: 21389074.
13. Dilsiz A, Kiliç N, Aydin T, Ates FN, Zihni M, Bulut C. Leptin levels in gingival crevicular fluid during orthodontic tooth movement. Angle Orthod. 2010 May;80(3):504-8. doi: 10.2319/072109-402.1. PMID: 20050744.
14. Domínguez A, Gómez C, Palma JC. Effects of low-level laser therapy on orthodontics: rate of tooth movement, pain, and release of RANKL and OPG in GCF. Lasers Med Sci. 2015 Feb;30(2):915-23. doi: 10.1007/s10103-013-1508-x. Epub 2013 Dec 18. PMID: 24346335.
15. Drummond S, Canavarro C, Perinetti G, Teles R, Capelli J Jr. The monitoring of gingival crevicular fluid volume during orthodontic treatment: a longitudinal randomized split-mouth study. Eur J Orthod. 2012 Feb;34(1):109-13. doi: 10.1093/ejo/cjq172. Epub 2011 Jan 27. PMID: 21273285.
16. Dudic A, Kiliaridis S, Mombelli A, Giannopoulou C. Composition changes in gingival crevicular fluid during orthodontic tooth movement: comparisons between tension and compression sides. Eur J Oral Sci. 2006 Oct;114(5):416-22. doi: 10.1111/j.1600-0722.2006.00387.x. PMID: 17026508.

17. Ford H, Suri S, Nilforoushan D, Manolson M, Gong SG. Nitric oxide in human gingival crevicular fluid after orthodontic force application. Arch Oral Biol. 2014 Nov;59(11):1211-6. doi: 10.1016/j.archoralbio.2014.07.015. Epub 2014 Aug 9. PMID: 25108338.
18. Genc G, Kocadereli I, Tasar F, Kilinc K, El S, Sarkarati B. Effect of low-level laser therapy (LLLT) on orthodontic tooth movement. Lasers Med Sci. 2013 Jan;28(1):41-7. doi: 10.1007/s10103-012-1059-6. Epub 2012 Feb 18. PMID: 22350425.
19. Giannopoulou C, Mombelli A, Tsinidou K, Vasdekis V, Kamma J. Detection of gingival crevicular fluid cytokines in children and adolescents with and without fixed orthodontic appliances. Acta Odontol Scand. 2008 Jun;66(3):169-73. doi: 10.1080/00016350802123126. PMID: 18568476.
20. Grant M, Wilson J, Rock P, Chapple I. Induction of cytokines, MMP9, TIMPs, RANKL and OPG during orthodontic tooth movement. Eur J Orthod. 2013 Oct;35(5):644-51. doi: 10.1093/ejo/cjs057. Epub 2012 Sep 17. PMID: 22987319.
21. Grieve WG 3rd, Johnson GK, Moore RN, Reinhardt RA, DuBois LM. Prostaglandin E (PGE) and interleukin-1 beta (IL-1 beta) levels in gingival crevicular fluid during human orthodontic tooth movement. Am J Orthod Dentofacial Orthop. 1994 Apr;105(4):369-74. doi: 10.1016/s0889-5406(94)70131-8. PMID: 8154462.
22. Hoshino-Itoh J, Kurokawa A, Yamaguchi M, Kasai K. Levels of t-PA and PAI-2 in gingival crevicular fluid during orthodontic tooth movement in adults. Aust Orthod J. 2005 May;21(1):31-7. PMID: 16433079.
23. Ingman T, Apajalahti S, Mäntylä P, Savolainen P, Sorsa T. Matrix metalloproteinase-1 and -8 in gingival crevicular fluid during orthodontic tooth movement: a pilot study during 1 month of follow-up after fixed appliance activation. Eur J Orthod. 2005 Apr;27(2):202-7. doi: 10.1093/ejo/cjh097. PMID: 15817630.
24. Insee K, Pothacharoen P, Kongtawelert P, Ongchai S, Jotikasthira D, Krisanaprakornkit S. Comparisons of the chondroitin sulphate levels in orthodontically moved canines and the clinical outcomes between two different force magnitudes. Eur J Orthod. 2014 Feb;36(1):39-46. doi: 10.1093/ejo/cjs109. Epub 2013 Mar 26. PMID: 23535117.
25. Intachai I, Krisanaprakornkit S, Kongtawelert P, Ong-chai S, Buranastidporn B, Suzuki EY, Jotikasthira D. Chondroitin sulphate (WF6 epitope) levels in peri-miniscrew implant crevicular fluid during orthodontic loading. Eur J Orthod. 2010 Feb;32(1):60-5. doi: 10.1093/ejo/cjp056. Epub 2009 Sep 13. PMID: 19752017.
26. Iwasaki LR, Chandler JR, Marx DB, Pandey JP, Nickel JC. IL-1 gene polymorphisms, secretion in gingival crevicular fluid, and speed of human orthodontic tooth movement. Orthod Craniofac Res. 2009 May;12(2):129-40. doi: 10.1111/j.1601-6343.2009.01446.x. PMID: 19419456.
27. Iwasaki LR, Haack JE, Nickel JC, Reinhardt RA, Petro TM. Human interleukin-1 beta and interleukin-1 receptor antagonist secretion and velocity of tooth movement. Arch Oral Biol. 2001 Feb;46(2):185-9. doi: 10.1016/s0003-9969(00)00088-1. PMID: 11163326.
28. Kawasaki K, Takahashi T, Yamaguchi M, Kasai K. Effects of aging on RANKL and OPG levels in gingival crevicular fluid during orthodontic tooth movement. Orthod Craniofac Res. 2006 Aug;9(3):137-42. doi: 10.1111/j.1601-6343.2006.00368.x. PMID: 16918678.
29. Kuroki H, Miyagawa Y, Shimomura-Kuroki J, Endo T, Shimomura H. Identification of marker proteins by orthodontic treatment: relationship of RANKL in the gingival crevicular fluid and of amylase in whole saliva with orthodontic treatment. Odontology. 2014 Jul;102(2):303-9. doi: 10.1007/s10266-013-0121-6. Epub 2013 Jul 18. PMID: 23864436.
30. Last KS, Donkin C, Embery G. Glycosaminoglycans in human gingival crevicular fluid during orthodontic movement. Arch Oral Biol. 1988;33(12):907-12. doi: 10.1016/0003-9969(88)90021-0. PMID: 3076754.
31. Luppanapornlarp S, Kajii TS, Surarit R, Iida J. Interleukin-1beta levels, pain intensity, and tooth movement using two different magnitudes of continuous orthodontic force. Eur J Orthod. 2010 Oct;32(5):596-601. doi: 10.1093/ejo/cjp158. Epub 2010 Jun 9. PMID: 20534713.
32. Nishijima Y, Yamaguchi M, Kojima T, Aihara N, Nakajima R, Kasai K. Levels of RANKL and OPG in gingival crevicular fluid during orthodontic tooth movement and effect of compression force on releases from periodontal ligament cells in vitro. Orthod Craniofac Res. 2006 May;9(2):63-70. doi: 10.1111/j.1601-6343.2006.00340.x. PMID: 16764680.
33. Pender N, Samuels RH, Last KS. The monitoring of orthodontic tooth movement over a 2-year period by analysis of gingival crevicular fluid. Eur J Orthod. 1994 Dec;16(6):511-20. doi: 10.1093/ejo/16.6.511. PMID: 7720796.

34. Perinetti G, Serra E, Paolantonio M, Bruè C, Meo SD, Filippi MR, Festa F, Spoto G. Lactate dehydrogenase activity in human gingival crevicular fluid during orthodontic treatment: a controlled, short-term longitudinal study. J Periodontol. 2005 Mar;76(3):411-7. doi: 10.1902/jop.2005.76.3.411. PMID: 15857076.
35. Rauten AM, Surlin P, Oprea B, Siloşi I, Moisa M, Caramizaru D, Vătu M. Matrix metalloproteinase 9 levels in gingival crevicular fluid in patients after periodontal microsurgery for orthodontic induced gingival hypertrophy. Rom J Morphol Embryol. 2011;52(1 Suppl):431-3. PMID: 21424088.
36. Ren Y, Maltha JC, Van't Hof MA, Von Den Hoff JW, Kuijpers-Jagtman AM, Zhang D. Cytokine levels in crevicular fluid are less responsive to orthodontic force in adults than in juveniles. J Clin Periodontol. 2002 Aug;29(8):757-62. doi: 10.1034/j.1600-051x.2002.290813.x. PMID: 12390573.
37. Ribagin LS, Rashkova MR. Matrix metalloproteinase-8 and interleukin-1beta in gingival fluid of children in the first three months of orthodontic treatment with fixed appliances. Folia Med (Plovdiv). 2012 Jul-Sep;54(3):50-6. doi: 10.2478/v10153-011-0097-3. PMID: 23270207.
38. Rody WJ Jr, Wijegunasinghe M, Wiltshire WA, Dufault B. Differences in the gingival crevicular fluid composition between adults and adolescents undergoing orthodontic treatment. Angle Orthod. 2014 Jan;84(1):120-6. doi: 10.2319/012813-85.1. Epub 2013 May 21. PMID: 23687969.
39. Samuels RH, Pender N, Last KS. The effects of orthodontic tooth movement on the glycosaminoglycan components of gingival crevicular fluid. J Clin Periodontol. 1993 May;20(5):371-7. doi: 10.1111/j.1600-051x.1993.tb00375.x. PMID: 8501278.
40. Sari E, Uçar C. Interleukin 1beta levels around microscrew implants during orthodontic tooth movement. Angle Orthod. 2007 Nov;77(6):1073-8. doi: 10.2319/100506-405.1. PMID: 18004920.
41. Serra E, Perinetti G, D'Attilio M, Cordella C, Paolantonio M, Festa F, Spoto G. Lactate dehydrogenase activity in gingival crevicular fluid during orthodontic treatment. Am J Orthod Dentofacial Orthop. 2003 Aug;124(2):206-11. doi: 10.1016/s0889-5406(03)00407-4. PMID: 12923518.
42. Sugiyama Y, Yamaguchi M, Kanekawa M, Yoshii M, Nozoe T, Nogimura A, Kasai K. The level of cathepsin B in gingival crevicular fluid during human orthodontic tooth movement. Eur J Orthod. 2003 Feb;25(1):71-6. doi: 10.1093/ejo/25.1.71. PMID: 12608726.
43. Surlin P, Silosi I, Rauten AM, Cojocaru M, Foia L. Involvement of TSP1 and MMP9/NGAL in angiogenesis during orthodontic periodontal remodeling. ScientificWorldJournal. 2014;2014:421029. doi: 10.1155/2014/421029. Epub 2014 May 20. PMID: 24967433; PMCID: PMC4054803.
44. Teng GY, Liou EJ. Interdental osteotomies induce regional acceleratory phenomenon and accelerate orthodontic tooth movement. J Oral Maxillofac Surg. 2014 Jan;72(1):19-29. doi: 10.1016/j.joms.2013.09.012. PMID: 24331564.
45. Toia M, Galazzo R, Maioli C, Granata R, Scarlatti F. The IGF-I/IGFBP-3 system in gingival crevicular fluid and dependence on application of fixed force. J Endocrinol Invest. 2005 Dec;28(11):1009-14. doi: 10.1007/BF03345340. PMID: 16483180.
46. Toygar HU, Kircelli BH, Bulut S, Sezgin N, Tasdelen B. Osteoprotegerin in gingival crevicular fluid under long-term continuous orthodontic force application. Angle Orthod. 2008 Nov;78(6):988-93. doi: 10.2319/100507-483.1. PMID: 18947279.
47. Tzannetou S, Efstratiadis S, Nicolay O, Grbic J, Lamster I. Comparison of levels of inflammatory mediators IL-1beta and betaG in gingival crevicular fluid from molars, premolars, and incisors during rapid palatal expansion. Am J Orthod Dentofacial Orthop. 2008 May;133(5):699-707. doi: 10.1016/j.ajodo.2006.03.044. PMID: 18456143.
48. Uematsu S, Mogi M, Deguchi T. Interleukin (IL)-1 beta, IL-6, tumor necrosis factor-alpha, epidermal growth factor, and beta 2-microglobulin levels are elevated in gingival crevicular fluid during human orthodontic tooth movement. J Dent Res. 1996 Jan;75(1):562-7. doi: 10.1177/00220345960750010801. PMID: 8655760.
49. Waddington RJ, Embery G, Samuels RH. Characterization of proteoglycan metabolites in human gingival crevicular fluid during orthodontic tooth movement. Arch Oral Biol. 1994 May;39(5):361-8. doi: 10.1016/0003-9969(94)90165-1. PMID: 8060258.
